# Salivary Metabolomics of Well and Poorly Controlled Type 1 and Type 2 Diabetes

**DOI:** 10.1155/2022/7544864

**Published:** 2022-08-24

**Authors:** Sompop Bencharit, James Carlson, Warren C. Byrd, Escher L. Howard-Williams, Jackson T. Seagroves, Susan McRitchie, John B. Buse, Susan Sumner

**Affiliations:** ^1^Department of Oral and Craniofacial Molecular Biology, School of Dentistry, Department of Biomedical Engineering, Virginia Commonwealth University, Richmond, VA, USA; ^2^Systems and Translational Sciences, RTI International, Research Triangle Park, Chapel Hill, NC, USA; ^3^School of Dentistry, University of North Carolina, Chapel Hill, NC, USA; ^4^Nutritional Research Institute, University of North Carolina, Chapel Hill, NC, USA; ^5^Department of Internal Medicine, School of Medicine, University of North Carolina, Chapel Hill, NC, USA

## Abstract

**Objective:**

The concentrations of endogenous metabolites in saliva can be altered based on the systemic condition of the hosts and may, in theory, serve as a reflection of systemic disease progression. Hemoglobin A1C is used clinically to measure long-term average glycemic control. The aim of the study was to demonstrate if there were differences in the salivary metabolic profiles between well and poorly controlled type 1 and type 2 subjects with diabetes. *Subjects and Methods*. Subjects with type 1 and type 2 diabetes were enrolled (*n* = 40). The subjects were assigned to phenotypic groups based on their current level of A1C: <7 = well-controlled and >7 = poorly controlled. Demographic data, age, gender, and ethnicity, were used to match the two phenotypic groups. Whole saliva samples were collected and immediately stored at −80°C. Samples were spiked using an isotopically labeled internal standard and analyzed by UPLC-TOF-MS using a Waters SYNAPT G2-Si mass spectrometer.

**Results:**

Unsupervised principal components analysis (PCA) and orthogonal partial least squares regression discrimination analysis (OPLS-DA) were used to define unique metabolomic profiles associated with well and poorly controlled diabetes based on A1C levels.

**Conclusion:**

OPLS-DA demonstrates good separation of well and poorly controlled in both type 1 and type 2 diabetes. This provides evidence for developing saliva-based monitoring tools for diabetes.

## 1. Introduction

Saliva is a readily available biological fluid that can be obtained noninvasively with moderate training and is cost-effective for screening large populations [1]. Saliva's chief advantages over other biospecimens include that it is easy to collect, ease of collection, minimally invasive for patients and study participants who may be adverse to blood collection, and achievable in study sites where more invasive samples are not safe or feasible. Saliva contains an array of proteins and metabolites that are secreted from salivary glands, oral mucosa, and gingival crevice fluid (GCF), as well as from the upper respiratory tract and possibly the gastrointestinal tract [2]. Proteins and metabolites may differ based on the gland saliva is collected from, and systemic conditions such as cancer, HIV, and periodontal disease each manifest differently in an individual's salivary profile [1, 3–6]. Over the past 2-3 decades, attempts have been made to measure salivary transcriptomes, proteomes, and metabolomes in order to identify differences between healthy and diseased subjects [7–11]. It is important to emphasize that inflammation can link systemic changes to oral conditions. A systematic review suggested that there is a link between patients' oral conditions, in particular their microbiome, and systemic condition [12]. Oral-systemic connections can be seen in periodontal disease [13]. Upregulation of inflammatory markers or cytokines can be linked to the activation of nuclear factor-kappa B (NF-*κ*B) via upregulation of proteins such as transglutaminase 2 [14]. An early detection of a certain biomarker can be reflective of oral and systemic conditions [14].

For patients suffering from diabetes mellitus, maintaining one's glucose level can reduce the risk of diabetes-related complications and improve quality of life [15]. Glucose levels in people with diabetes have traditionally been assessed by measuring hemoglobin A1c (HbA1c) levels, which serve as a proxy for average glucose levels over the past 3 months, the typical red blood cell lifetime [15, 16]. The HbA1c level is used to diagnose and monitor progression of diabetes; diabetes is defined as having HbA1c levels of ≥6.5% [17]. Among people with diabetes, the level of disease control can be stratified into two groups: poorly controlled diabetes, defined as having an HbA1c level of ≥9.5%, and well-controlled diabetes, defined as having an HbA1c level of ≤7.5% [18].

Our group along with others recently showed that significant proteomic differences exist in people with diabetes as compared to healthy controls [19–22]. More importantly, salivary proteomes appear to be altered based on systemic changes from healthy to prediabetics to full diabetes [19]. We also found changes in salivary proteomes in type 1 and type 2 diabetes based on their A1C values [20]. Similar to proteomics, methods in metabolomics have recently been introduced to define salivary metabolites. Salivary metabolites and metabolomic changes are associated with several diseases, including multiple types of cancers and periodontal disease [6, 23]. Levels of salivary metabolites have been associated with diabetes development [24]. However, little is known about the differences in the salivary metabolomics profiles of type 1 and type 2 diabetes based on the level of glycemic control measured by HbA1c.

Here, for the first time we provide a comparison of metabolomics profiles of well and poorly controlled type 1 and type 2 diabetes. The rationale for the study was that the changes in the HbA1c levels would trigger changes in serum/plasma, which in turn affect the salivary metabolomic profiles, possibly through secondary mechanisms such as inflammatory responses. This pilot study provides promising supportive evidence for saliva as a diagnostic fluid for diabetic progression and treatment responses.

## 2. Materials and Methods

### 2.1. Subject Recruitment and Sample Collection

The study protocol was approved by the Office of Human Research Ethics, the University of North Carolina (UNC) Institutional Review Board (IRB), No. 10–0492. All subjects were recruited from the UNC Diabetes Care Center. The inclusion criteria included patients who were between the ages of 18 and 89 years, had been diagnosed with type 1 or type 2 diabetes, had come for a regular visit at the diabetes care clinic, and had HbA1c tested at the visit. The exclusion criteria included patients who were nondiabetic, patients who were younger than 18 or older than 89, and patients who refused to provide saliva samples. All subjects gave written informed consent for saliva sample collection, storage, and analysis for this study. Study participants' names and other HIPAA identifiers were removed from this report. No identified information in this work can be traced back to any study participant. The saliva collection protocol was described previously [20, 21]. About 2 hours before sample collection, subjects were asked to refrain from drinking, eating, and practicing any oral hygiene habits. The sample collection was done between 9 am and 12 pm and before lunchtime. The subject was asked to spit into a 15-ml falcon tube within 30–60 minutes to provide about 4 ml of saliva samples. The falcon tube was then placed in a centrifuge at 4,000 rpm at 4°C for 15 minutes to remove food debris. About 750 ul of the supernatant was then aliquoted into a 1.5 ml cryotube. The tube was then fast frozen in liquid nitrogen and placed at −80°C for storage prior to analysis [20, 21].

### 2.2. Broad-Spectrum Metabolomics

This study was conducted using methods similar to those previously described [25, 26]. A 100 *µ*L aliquot of each study sample's saliva was used for analysis. In addition, a small aliquot of each study sample extract was pooled to create a total pool sample. Total pool samples were prepared identically to the individual study samples and were analyzed at fixed intervals across the run. All study samples were randomized for extraction. A cold methanol with tryptophan-d5 internal standard (500 *µ*l) was added to each study and pooled the sample for protein precipitation. After centrifugation at 16,000 rcf, 450 *µ*l of supernatant was lyophilized overnight and reconstituted in 100 *µ*l of 95 : 5 water: methanol for broad-spectrum metabolomics analysis.

Samples were analyzed on an SYNAPT G2-Si QTOF mass spectrometer coupled to an ACQUITY UPLC (Waters Corporation, MA). Prior to analyzing the study samples, the column and the system were equilibrated with ten injections of QC samples. 5 *µ*l of the salivary extract was injected for mass spectrometric analysis. Study samples were randomized, and QC pool samples were inserted in the analytical run sequence following the injection of 3–5 study samples. The compounds were separated on a Waters ACQUITY BEH HSS T3 column (2.1 × 100 mm, 1.8 *µ*m particle size) operating at 50°C using a reversed-phase chromatographic method. A gradient mobile phase consisting of water with 0.1% formic acid (A) and methanol with 0.1% formic acid (B) was used as previously described [27]. All MS data were collected over 50–1000 m/z in ESI-positive and negative ion modes using an MS^E^ data acquisition method. Leucine enkephalin was used as the lock mass calibrant and a lock mass scan was collected every 45 s and averaged over 3 scans to perform mass correction. Source and desolvation temperatures were set at 110°C and 450°C, respectively.

Unsupervised data analysis was first performed to see the overall trend, pattern, and outliers. Later, to classify potential biomarker metabolites, labeled-biomarker supervised data analysis was performed. The raw mass spectrometric data were processed (alignment, normalization, and peak picking) using Progenesis QI (Waters Corporation). Multivariate analysis (PCA and OPLS-DA) of the metabolomics data was performed using SIMCA (Umetrics, Umea, Sweden) [28]. A putative identification of the signals differentiating the study groups was made using a database search against RTI-RCMRC's in-house exact-mass-retention-time library of standards (890 compounds) and the Human Metabolome Database (HMDB) release version 3.60.

## 3. Results

### 3.1. Subjects' Demographics

All subjects were matched by age, gender, and ethnicity (Table 1). Note that in this exploratory study, we only matched Caucasian subjects since the number of subjects in other ethnicities was too small and could not be matched. The HbA1c cutoff of 7 was used to define well or poor glycemic control. However, the average HbA1c in well-controlled was below 6.5 and the average HbA1c in poorly controlled was higher than 9 (Table 1).

### 3.2. Analysis of Saliva Metabolomics

**Figure 1:**
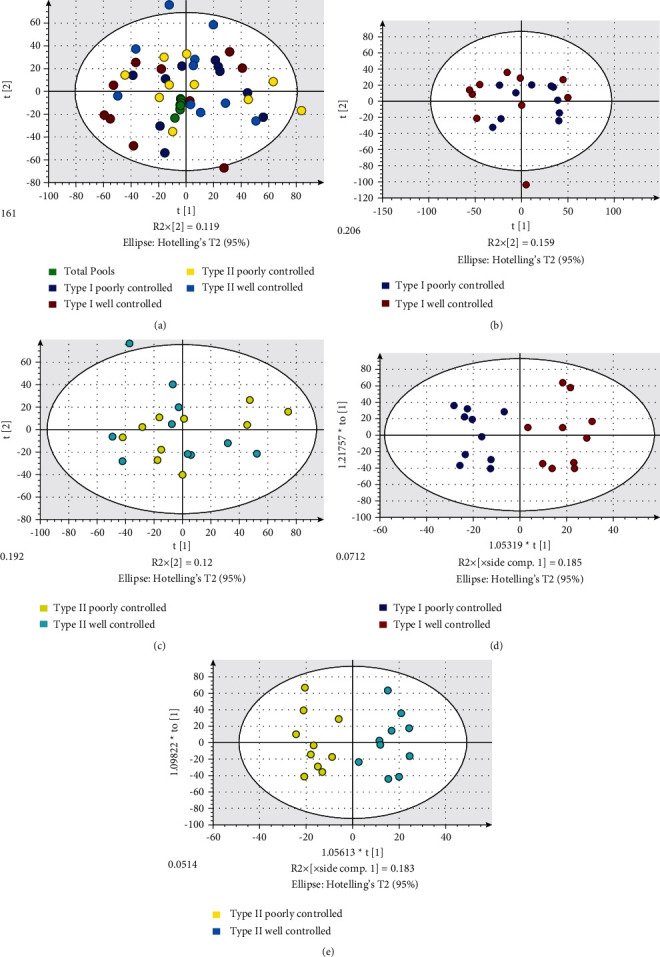
Salivary metabolomics for diabetes. (a) Unsupervised multivariate analysis (PCA) of type I and II diabetes salivary samples with pooled quality control samples tightly clustered and centered; unsupervised PCA analysis did not differentiate poorly and well controlled type I (b) and II (c) diabetes salivary samples; (d) supervised analysis (OPLS-DA) of type I diabetes (d) and type II (e) saliva samples differentiated these poorly and well controlled subjects saliva samples.

Saliva samples were spiked with an isotopically labeled internal standard. Each study sample was extracted with methanol and lyophilized and randomized with quality control samples prior to analysis by UPLC-TOF-MS on a Waters SYNAPT G2-Si mass spectrometer [22, 23]. The data were processed using Progenesis Qi Software, and multivariate analysis was conducted using SIMCA 14. Unsupervised principal component analysis (PCA) was first used to evaluate the quality of the analysis by demonstrating that the QC pool samples are tightly clustered and centered in the middle of the samples from where they were derived (Figure1(a)). Unsupervised PCA analysis did not reveal good differentiation between the well and poorly controlled diabetes (type 1, Figure 1(b); type 2, Figure 1(c)). A supervised method (Orthogonal Projections to Latent Structures Discriminant Analysis, OLPS-DA) was used to examine possible relationships among the metabolites in each type of diabetes. OLPS-DA could differentiate the well and poorly controlled groups (Figures 1(d) and 1(e)) creating a signature of signals that best define these groups. Tables 2 and 3 demonstrate the biomarker metabolites differentially expressed in poorly controlled vs. well-controlled type I and type II diabetes, respectively.

## 4. Discussion

The results demonstrated, for the first time, the salivary metabolomic profiles of type 1 and type 2 diabetes based on different levels of HbA1c levels. This is an important finding highlighting the possibility of using salivary metabolites to monitor diabetic conditions. Advancements in the fields of serum proteomic and metabolomic profiling have afforded the use of blood as a diagnostic tool for diabetes [24, 29–31]. However, the use of saliva as a diabetes diagnostic tool has been minimally researched. Previously, we have demonstrated that patients with type 1 and type 2 diabetes have a significantly different salivary proteomic profile based on HbA1c levels. [20] In addition, we have shown that patients with diabetes have significantly higher levels of diabetes-related inflammatory biomarkers and lower levels of other biomarkers [21]. These salivary biomarker profiles may mimic the changes found in serum. Previous literature regarding salivary proteomic differences in patients with diabetes is limited. In accordance with our previous study, Bencharit et al. [20] found a significant difference in biomarker profiles in both types of diabetes based on the subject's HbA1c, specifically with proteins involved in metabolism and immune response [32]. Additional research suggests that type 1 diabetes compared to controls has significantly higher levels of proinflammatory biomarkers [22]. In terms of salivary metabolomics, Barnes et al. demonstrated a difference in metabolite profiles for patients with type 2 diabetes compared to controls [33]. However, the difference in metabolomic profiles between “poorly” and “well-controlled” type 1 and type 2 diabetes has, to our knowledge, never been investigated. In this exploratory study, we have shown that type 1 and type 2 “poorly” and “well-controlled” diabetes can be differentiated based on salivary metabolomics profiles. Furthermore, these differences in salivary metabolomic profiles may suggest biochemical processes are different in people with diabetes based on the level of glycemic control.

The main limitation of this study was the small study population used. Current research into salivary metabolomics in people with diabetes using a large sample size is nonexistent. We also limited our study to the Caucasian population to minimize variability. Previous research has used sample sizes of less than 50, which exacerbates the problem of variability between individuals' salivary protein profiles. Thus, the results of this study and other similar studies are limited in applicability until large-scale patient populations are studied. In addition, metabolites in serum are usually highly regulated within a certain range in the population. Thus, serum metabolomics profiling is reproducible and has successfully been applied to the diagnosis of Alzheimer's disease, bipolar disorder, autism, and schizophrenia [34]. On the contrary, the physiological ranges of salivary metabolites are much wider than those in serum. In addition, two healthy subjects can have different baseline values for each salivary metabolite. This presents challenges in reproducibility due to subject-to-subject metabolomic profile variability. It is also important to note here that we use A1C = 1 as a cutoff to define well (A1C < 7) and poorly (A1C > 7) controlled diabetes to select the subjects. However, in the well-controlled groups, the A1C is 6.31 ± 0.29 and in the poorly controlled group, the A1C is 9.44 ± 0.78. The values are aligned with other clinical recommendations and studies [35, 36]. The A1C of 6.5 or lower represents a low risk or well-controlled diabetes [35], whereas A1C > 9 represents poorly controlled diabetes [36].

Orthogonal Projections to Latent Structures Discriminant Analysis (OLPS-DA) is a supervised statistical technique that determines the best difference between the phenotypic groups, as opposed to the unsupervised principal component analysis (PCA). OPLS-DA ideally allows for improved biomarker discovery via differentiating groups and is useful when dealing with datasets with a large number of correlated variables such as metabolites [37]. Unlike PCA which considers each biomarker as an independent variable, OPLS-DA considers all variables have some interdependency. This results in a variable importance plot (VIP) value that indicates the variables' importance in differentiating the groups.

Future studies should include a replication of this study with a larger population. We propose to conduct an in-depth analysis to identify signals as salivary biomarkers associated with worsening glycemic control which may be related to diabetic-associated clinical symptoms or complications. We also propose to target certain salivary metabolites in patients with type 1 and type 2 diabetes to further develop novel noninvasive saliva-based analytical tools.

Measuring blood glucose levels can be painful, costly, and bothersome for people with diabetes. Identifying biomarkers in saliva associated with poor glycemic control may provide an alternative avenue for diabetes management. Good glycemic control may reduce diabetes-related complications and improve quality of life. Thus, a simpler method for measuring glycemic control would be an attractive opportunity for people with diabetes and health professionals alike. A device to quantitatively measure salivary biomarkers [9–11, 37] would allow for a simpler evaluation of an individual's glycemic control. An accurate, biomarker-specific, and cost-effective tool could revolutionize diabetes management by using saliva as a diagnostic tool.

## 5. Conclusion

Salivary metabolomic profiles can differentiate well-controlled low HbA1c levels from poorly controlled high HbA1c levels in both type 1 and type 2 diabetes. Saliva metabolites may in the future be developed as a noninvasive tool for monitoring diabetic-related glycemic control.

## Figures and Tables

**Table 1:** Demographic data of subjects.

Type	Glycemic control^*∗*^	Gender ratio (*n* = 10)	Age		A1C	
		Female/male	Average	Standard deviation	Average	Standard deviation
Type 1	Well-controlled	4/6	44.3	14.95	6.27	0.4
	Poorly controlled	4/6	45.9	21.14	10	2.25

Type 2	Well-controlled	4/6	59	7.7	6.31	0.29
	Poorly controlled	4/6	56.3	6.67	9.44	0.78

^*∗*^A cutoff point of A1C = 7 was used to determine glycemic control in this study.

**Table 2:** Summary of salivary metabolites differentially expressed in poorly controlled vs. well-controlled type I diabetes.

Metabolite compound ID	VIP	*P* value^*∗*^	Fold change^*∗∗*^
(1967 peaks)	0.1–2.4	0.001–1	−475, −733.6
(4Z)-4-(3,3-Dimethyltriazanylidene)-4H-imidazole-5-carboxamide	1.4	0.016	1.5
17*β*-Estradiol 3-benzoate	1.5	0.101	1.4
2-Isopropyl-4a-methyl-8-methylenedecahydro-1-naphthalenol	1.3	0.118	−3.8
2-Methyl-3-ketovaleric acid	1.2	0.302	−1.3
2-Oxo-4-methylthiobutanoic acid	1.4	0.044	1.7
5,6-Dihydrouridine	1.1	0.212	1.3
APGPR enterostatin	1.3	0.173	−2
Androsterone glucuronide	1.1	0.234	2
Coumarin	1.1	0.081	2.7
Cytidine monophosphate N-acetylneuraminic acid	1.4	0.359	−1.6
D-Arginyl-L-arginyl-D-isoleucine	1.1	0.308	−1.3
DG (14:1/25:0/0:0)	1.4	0.177	−1.9
DL-Glutamine	2	0.051	1.2
DL-Asparagine	1.6	0.062	−1.5
Deoxypyridinoline	1.4	0.136	1.5
Dopamine	1	0.91	1
Edoxudine	1.7	0.026	−1.3
GPA (4:0/18:3)	1.6	0.068	1.5
GPSer (9:0/12:0)	1.8	0.049	2.1
Glycyl-phenylalanine	1.2	0.434	1.2
Glycylproline	1.8	0.086	1.3
L-Acetylcarnitine	1.1	0.567	−1.2
L-Proline	1.5	0.105	1.4
N-Acetyl-L-phenylalanine	1.2	0.761	1.1
N-Glycolylneuraminic acid	1.7	0.041	2.7
N6-Acetyl-L-lysine	1	0.766	−1.1
Ne, Ne dimethyllysine	1	0.927	1
PE (24:0/18:2 (9Z,12Z))	1.9	0.044	−2.3
Paramethasone	1.1	0.578	1.2
Salicylic acid	1	0.268	1.7
Stearic acid	1.7	0.037	−1.7
Tyrosyl-proline	1.8	0.015	1.9

^*∗*^ *t*-test with Satterthwaite correction. ^*∗∗*^A positive fold change indicates mean of poorly controlled > mean of well controlled. VIP, variable influence on projection.

**Table 3:** Summary of salivary metabolites differentially expressed in poorly controlled vs. well-controlled type II diabetes.

Metabolite compound ID	VIP	*P* value^*∗*^	Fold change^*∗∗*^
1490 peaks	0.2–2.4	0.002–0.999	−107, −382
(2S)-3-Hydroxy-1,1-dimethyl-2-pyrrolidiniumcarboxylate	1.6	0.075	−1.5
(3*α*,4*β*,8*α*,12R)-15-Acetoxy-3,4-dihydroxy-12,13-epoxytrichothec-9-en-8-yl 3-methylbutanoate	1.2	0.792	1.1
(3beta)-Gonan-3-yl beta-D-glucopyranoside	1.5	0.302	−1.1
(4Z)-4-(3,3-Dimethyltriazanylidene)-4H-imidazole-5-carboxamide	1	0.344	96.7
(6E)-3,7,11-Trimethyl-6,10-dodecadien-1-yl dihydrogen phosphate	1.4	0.211	−1.2
2,3-Dinor-6-keto-prostaglandin F1 a	1.4	0.364	−1.2
2-Isopropyl-4a-methyl-8-methylenedecahydro-1-naphthalenol	1.5	0.117	10
2-Oxo-4-methylthiobutanoic acid	1.9	0.093	−1.4
3-Hydroxycoumarin	1.3	0.427	−1.1
3-Hydroxybenzoic acid	1.6	0.267	−1.3
3-Hydroxyhexadecanoylcarnitine	1.4	0.362	−1.2
8-Isoprostane	1.6	0.159	−1.2
Androsterone glucuronide	1.3	0.616	−1.1
Arginyl-aspartate	2.1	0.023	−1.4
Coumarin	2	0.038	−3.1
DL-Serine	1.7	0.07	−1.4
Deoxypyridinoline	1.6	0.054	1.4
Endomorphin-2	1.2	0.734	1.1
GPA (4:0/18:3)	1.2	0.638	−1.1
Hippuric acid	1.6	0.108	−1.3
L-Valine	2.1	0.037	−1.3
Lauramide	1.4	0.339	−1.1
LysoPE (0:0/16:0)	1.1	0.902	−1
Lysyl-asparagine	1.3	0.351	−1.2
MG (11:0/0:0/0:0)	1.5	0.154	−1.4
MGDG (20:5/14:1)	1.6	0.236	−1.2
MGDG (4:0/5:0)	2.3	0.013	−1.5
Methylimidazoleacetic acid	1.9	0.034	1.7
N-Glycolylneuraminic acid	1.2	0.264	2.3
N-Methylserotonin	2.1	0.016	−1.5
N6,N6,N6-Trimethyl-L-lysine	1.6	0.118	−1.6
Neurine	1.2	0.923	1
Pyrimidine	1.8	0.043	1.8
Pyrrolidine	1.2	0.587	−1.2
Saccharopine	1.9	0.065	−1.3
Salicylic acid	1.4	0.17	−1.3
Stearic acid	1.6	0.071	2.4
Ureidosuccinic acid	1.5	0.168	−1.3
Lauryl sulfate	1.6	0.105	−1.4

^*∗*^ *t*-test with Satterthwaite correction. ^*∗∗*^A positive fold change indicates mean of poorly controlled > mean of well-controlled.

## Data Availability

The data used to support the findings of this study are available from the corresponding author upon request.

## References

[1] Pfaffe T., Cooper-White J., Beyerlein P., Kostner K., Punyadeera C. (2011). Diagnostic potential of saliva: current state and future applications. *Clinical Chemistry*.

[2] Kaufman E., Lamster I. B. (2002). The diagnostic applications of saliva—a review. *Critical Reviews in Oral Biology & Medicine*.

[3] Hofman L. F. (2001). Human saliva as a diagnostic specimen. *Journal of Nutrition*.

[4] Schramm W., Angulo G. B., Torres P. C., Burgess-Cassler A. (1999). A simple saliva-based test for detecting antibodies to human immunodeficiency virus. *Clinical and Diagnostic Laboratory Immunology*.

[5] Tóthová L., Kamodyová N., Červenka T., Celec P. (2015). Salivary markers of oxidative stress in oral diseases. *Frontiers in Cellular and Infection Microbiology*.

[6] Trezzi J.-P., Vlassis N., Hiller K. (2015). The role of metabolomics in the study of cancer biomarkers and in the development of diagnostic tools. *Advances in Experimental Medicine and Biology*.

[7] Al-Tarawneh S. K., Border M. B., Dibble C. F., Bencharit S. (2011). Defining salivary biomarkers using mass spectrometry-based proteomics: a systematic review. *OMICS: A Journal of Integrative Biology*.

[8] Giannobile W. V., Beikler T., Kinney J. S., Ramseier C. A., Morelli T., Wong D. T. (2009). Saliva as a diagnostic tool for periodontal disease: current state and future directions. *Periodontology 2000*.

[9] Gardner A., Carpenter G., So P.-W. (2020). Salivary metabolomics: from diagnostic biomarker discovery to investigating biological function. *Metabolites*.

[10] Hyvärinen E., Savolainen M., Mikkonen J. J. W., Kullaa A. M. (2021). Salivary metabolomics for diagnosis and monitoring diseases: challenges and possibilities. *Metabolites*.

[11] Dame Z. T., Aziat F., Mandal R. (2015). The human saliva metabolome. *Metabolomics*.

[12] Martellacci L., Quaranta G., Patini R., Isola G., Gallenzi P., Masucci L. (2019). A Literature Review of Metagenomics and Culturomics of the Peri-implant Microbiome: Current Evidence and Future Perspectives. *Materials*.

[13] Matarese G., Isola G., Ramaglia L. (2016). Periodontal biotype: characteristic, prevalence and dimensions related to dental malocclusion. *Minerva Stomatologica*.

[14] Matarese G., Currò M., Isola G. (2015). Transglutaminase 2 up-regulation is associated with RANKL/OPG pathway in cultured HPDL cells and THP-1-differentiated macrophages. *Amino Acids*.

[15] Skyler J. S. (2004). Effects of glycemic control on diabetes complications and on the prevention of diabetes. *Clinical Diabetes*.

[16] Rodbard H. W., Jellinger P. S., Davidson J. A. (2009). Statement by an American association of clinical endocrinologists/American college of endocrinology consensus panel on type 2 diabetes mellitus: an algorithm for glycemic control. *Endocrine Practice*.

[17] The International Expert Committee (2009). International expert committee report on the role of the A1C assay in the diagnosis of diabetes. *Diabetes Care*.

[18] Shani M., Taylor T. R., Vinker S. (2008). Characteristics of diabetics with poor glycemic control who achieve good control. *The Journal of the American Board of Family Medicine*.

[19] Rao P. V., Reddy A. P., Lu X. (2009). Proteomic identification of salivary biomarkers of type-2 diabetes. *Journal of Proteome Research*.

[20] Bencharit S., Baxter S. S., Carlson J. (2013). Salivary proteins associated with hyperglycemia in diabetes: a proteomic analysis. *Molecular BioSystems*.

[21] Border M. B., Schwartz S., Carlson J. (2012). Exploring salivary proteomes in edentulous patients with type 2 diabetes. *Molecular BioSystems*.

[22] Cabras T., Pisano E., Mastinu A. (2010). Alterations of the salivary secretory peptidome profile in children affected by type 1 diabetes. *Molecular and Cellular Proteomics*.

[23] Mikkonen J. J. W., Singh S. P., Herrala M., Lappalainen R., Myllymaa S., Kullaa A. M. (2016). Salivary metabolomics in the diagnosis of oral cancer and periodontal diseases. *Journal of Periodontal Research*.

[24] Wang T. J., Larson M. G., Vasan R. S. (2011). Metabolite profiles and the risk of developing diabetes. *Nature Medicine*.

[25] Dhungana S., Carlson J. E., Pathmasiri W. (2016). Impact of a western diet on the ovarian and serum metabolome. *Maturitas*.

[26] Milner J. J., Stewart D. A., Rebeles J. (2015). Obesity increases mortality and modulates the Lung metabolome during pandemic H1N1 influenza virus infection in mice. *The Journal of Immunology*.

[27] Dunn W. B., Broadhurst D., Begley P. (2011). Procedures for large-scale metabolic profiling of serum and plasma using gas chromatography and liquid chromatography coupled to mass spectrometry. *Nature Protocols*.

[28] Trygg J., Holmes E., Lundstedt T. (2007). Chemometrics in metabonomics. *Journal of Proteome Research*.

[29] Fiehn O., Garvey W. T., Newman J. W., Lok K. H., Hoppel C. L., Adams S. H. (2010). Plasma metabolomic profiles reflective of glucose homeostasis in non-diabetic and type 2 diabetic obese african-American women. *PLoS One*.

[30] Felig P., Marliss E., Cahill G. F. (1969). Plasma amino acid levels and insulin secretion in obesity. *New England Journal of Medicine*.

[31] Griffin J. L., Nicholls A. W. (2006). Metabolomics as a functional genomic tool for understanding lipid dysfunction in diabetes, obesity and related disorders. *Pharmacogenomics*.

[32] Floegel A., Stefan N., Yu Z. (2013). Identification of serum metabolites associated with risk of type 2 diabetes using a targeted metabolomic approach. *Diabetes*.

[33] Barnes V. M., Kennedy A. D., Panagakos F. (2014). Global metabolomic analysis of human saliva and plasma from healthy and diabetic subjects, with and without periodontal disease. *PLoS One*.

[34] Liu M., Zhou K., Li H. (2015). Potential of serum metabolites for diagnosing post-stroke cognitive impairment. *Molecular BioSystems*.

[35] Zhang X., Gregg E. W., Williamson D. F. (2010). A1C level and future risk of diabetes: a systematic review. *Diabetes Care*.

[36] Tsai C., Hayes C., Taylor G. W. (2002). Glycemic control of type 2 diabetes and severe periodontal disease in the US adult population. *Community Dentistry and Oral Epidemiology*.

[37] Vajargah K. F., Sadeghi-Bazargani H., Mehdizadeh-Esfanjani R., Savadi-Oskouei D., Farhoudi M. (2012). OPLS statistical model versus linear regression to assess sonographic predictors of stroke prognosis. *Neuropsychiatric Disease and Treatment*.

